# Functional implications of PABPC1 in the development of ovarian cancer

**DOI:** 10.1515/med-2021-0278

**Published:** 2021-05-14

**Authors:** Cong Feng, Yan-Hua Han, Na Qi, Jia Li, Qing-Hua Sheng, Yu Liu, Li-Li Yang

**Affiliations:** Department of Obstetrics and Gynecology, First Affiliated Hospital, Heilongjiang University of Chinese Medicine, Harbin, Heilongjiang 150040, People’s Republic of China; Department of Gynecology, Heilongjiang University of Chinese Medicine, Harbin, Heilongjiang 150040, People’s Republic of China; Department of Plastic and Maxillofacial Surgery, Heilongjiang Provincial Hospital, Harbin, Heilongjiang 150036, People’s Republic of China; Department of Urology, Heilongjiang Provincial Hospital, No. 82 Zhong-Shan Road, Xiang-Fang District, Harbin, Heilongjiang 150036, People’s Republic of China; Department of Gynecology, Hainan Provincial Hospital of Traditional Chinese Medicine, Haikou City, Hainan Province, People’s Republic of China

**Keywords:** PABPC1, ovarian carcinoma, proliferation, invasion and migration, EMT

## Abstract

This research aimed to probe the expression characteristics of poly(A)-binding protein cytoplasmic 1 (PABPC1) and its role on the phenotype of ovarian cancer (OC) cells and to further investigate the possible underlying mechanism. The expression of PABPC1 was analyzed according to the data from gene expression omnibus, The Cancer Genome Atlas (TCGA) and Oncomine databases and the RNA sequencing data set from TCGA were downloaded for evaluating the prognostic values. We revealed that compared with the healthy samples, PABPC1 was upregulated in OC samples. High expression of PABPC1 had a connection with a shorter survival for patients with OC. Loss and gain of function assays revealed that silencing PABPC1 significantly suppressed the viability, invasion and migration of SK-OV-3 cells, while PABPC1 overexpression in A2780 cells showed the reverse outcomes. Moreover, Western blot demonstrated that silencing PABPC1 notably inactivated the epithelial–mesenchymal transition (EMT) process, while upregulation of PABPC1 promoted the mitigation of epithelial phenotype and the acquisition of mesenchymal phenotype. Taken together, PABPC1 was upregulated in OC cells and served as a carcinogene to promote the OC cell growth and invasion partly by modulating the EMT process, which implied that PABPC1 might be considered as a useful biomarker for OC therapeutics.

## 1 Introduction

Ovarian cancer (OC), known as a common malignancy in gynecology, has the highest mortality among the malignant tumors in female reproductive system and seriously threatens women’s life and health [1,2]. The American Cancer Society estimated approximately 22,530 new cases of OC and 13,980 OC-related deaths in the United States in 2019 [3]. This carcinoma is frequently diagnosed at an advanced stage due to its asymptomatic development and the lack of effective diagnostic approach at an early stage [4,5]. Although patients with OC usually respond well to the first-line chemotherapy based on platinum compounds and taxanes, most of the patients with OC still relapse [6]. According to the platinum-free interval cutoff of 6 months, the first recurrence is usually classified as platinum sensitive and platinum resistant [7]. After this first recurrence, the patients are rarely cured, and the general approach used in this recurrence can guide the approach to subsequent recurrences [8]. Platinum-sensitive secondary recurrence permits the use of platinum-based chemotherapy and a further cytopenia surgery; however, the prognosis of these people is usually poor [9]. Nevertheless, in this scenario, the introduction of new targeted therapies changed the prognosis of patients with both platinum-sensitive and platinum-resistant recurrence. [9]. Therefore, it is of important significance for the treatment of OC to elucidate the potential mechanism of action of OC process.

Poly(A)-binding protein (PABP) family is usually considered as a protective barrier of the mRNA poly(A) tail to mediate multiple aspects of translation and stability of mRNA [10,11]. PABP cytoplasmic 1 (PABPC1) is widely distributed in the cytoplasm of eukaryotes, binds to the poly(A) tail and interacts with specific sequences in the mRNA, allowing it to play a vital role in many kinds of cellular activities such as participating in the initiation of translation and in the regulation of mRNA decay [12,13]. Years of research have demonstrated that PABPC1 was abnormally expressed in numerous tumors and was involved in carcinogenicity [14,15,16,17]. PABPC1 was found to be highly expressed in hepatocellular carcinoma and induced cell viability by promoting entry into the S phase [14]. It was also reported that PABPC1 plays a carcinogenic role by inhibiting the expression of miR-34c in gastric carcinoma [15]. However, low expression of PABPC1 could promote tumor cells growth and lead to a poor prognosis in esophageal cancer [16,17]. Herein the question that puzzled us is what role PABPC1 plays in OC, which has rarely been reported.

In view of this, aiming at investigating the role of PABPC1 in OC, we downloaded and analyzed the data from the online public databases and assessed the relationship between PABPC1 and survival rate. Aside from this, we detected the effects of PABPC1 on the phenotype of OC *in vitro* using the SK-OV-3 and A2780 cell lines. Furthermore, since accumulating studies have shown that the role of epithelial–mesenchymal transition (EMT) in OC is closely linked to the invasion and metastasis of tumor cells [18,19,20], the relationship between PABPC1 and EMT-related markers was also investigated. This work provided evidences *in vitro* supporting the possibility of PABPC1 serving as a carcinogene to enhance OC cells’ proliferative, invasive and migratory potential by modulating the EMT process.

## 2 Materials and methods

### 2.1 Data acquisition

Initially, we analyzed the expression of PABPC1 using the data set GSE54388 which was downloaded from Gene Expression Omnibus (GEO, https://www.ncbi.nlm.nih.gov/geo/) database. The RNA sequencing data sets were acquired from The Cancer Genome Atlas (TCGA, https://cancergenome.nih.gov) and normal samples were downloaded from Genotype-Tissue Expression (GTEx, https://gtexportal.org/home/) database. Different data sets including Bonome Ovarian data set and Yoshihara Ovarian data set from Oncomine (https://www.oncomine.org) database were also used to analyze the PABPC1 expression pattern. A total of 374 cases of OC in TCGA cohort were divided into high and low PABPC1 expression groups according to the median expression of PABPC1 for evaluating the correlation between PABPC1 and the overall survival rate.

### 2.2 Cell lines

Human OC cell lines SK-OV-3 and A2780 were obtained from the Shanghai Cell Bank, Chinese Academy of Medical Sciences (Shanghai, China), and OVCAR-3 was acquired from the American Type Culture Collection (Rockville, VA, USA). From Procell Technology (Wuhan, Hubei, China), normal OC cell line IOSE80 was purchased. All cells were incubated at 37°C under the 5% CO_2_ atmosphere in Roswell Park Memorial Institute-1640 medium (Procell) supplemented with 10% fetal bovine serum (FBS) and 1% penicillin–streptomycin solution.

### 2.3 RNA interference and transfection

Small interfering RNAs (siRNAs) targeting PABPC1 (si-PABPC1#1: 5′-CATCGACAATAAAGCACTAT-3′; si-PABPC1#2: 5′-CTAGCCAAATTGCTCAACTA-3′) and their control siRNA (si-con: 5′-CGAACTCACTGGTCTGACC-3′) were designed to downregulate PABPC1 expression. Plasmid pcDNA3.1-PABPC1 and empty vector pcDNA3.1 were conducted to overexpress PABPC1. All siRNA sequences and plasmid vectors were constructed by the GenePharma Corporation (Shanghai, China).

Lipofectamine™ 2000 transfection reagent (Thermo Fisher Scientific, Waltham, MA, USA) served to carry out transfection according to the manufacturer’s protocols. Upon transfection for 24 h, the transfection efficacy was detected.

### 2.4 RNA extraction and real-time quantitative polymerase chain reaction (RT-qPCR)

Using the TRIzol reagent (Invitrogen, Carlsbad, CA, USA), total RNAs from cells were extracted and then reverse transcribed by PrimeScript RT Reagent kit (TaKaRa, Tokyo, Japan). The mRNA level of PABPC1 was tested by RT-qPCR with the SYBR Premix Ex Taq (TaKaRa) on the Applied Biosystems 7500 Thermocycler (Thermo Fisher Scientific) according to the manufacturer’s protocols. Glyceraldehyde-3-phosphate dehydrogenase (GAPDH) served as the control. Primer sequences for RT-qPCR were:

PABPC1 forward: 5′-CAGAGAATGGCAAGTGTACGAGC-3′,

PABPC1 reverse: 5′-GCTAGGAGGATAGTATGCAGCAC-3′;

GAPDH forward: 5′-TGTGTCCGTCGTGGATCTGA-3′,

GAPDH reverse: 5′-CCTGCTTCACCACCTTCTTGA-3′.

### 2.5 Western blot

After transfection for 24 h, proteins were extracted from the cells using the radio immunoprecipitation assay lysis buffer (Beyotime, Nantong, China) on ice. Protein concentration was measured via the bicinchoninic acid protein assay kit (Beyotime). Equal amount of protein (20 μg) was separated by 10% sodium dodecyl sulfate–polyacrylamide gel electrophoresis and transferred onto polyvinylidene fluoride membranes, which were then blocked for an hour with 5% skimmed milk powder. Next the membranes were incubated with the primary antibodies including anti-PABPC1 (ab233280), anti-E-cadherin (ab1416), anti-N-cadherin (ab202030), anti-snail (ab229701) and anti-vimentin (ab193555) overnight at 4°C. Additionally, anti-PABPC1 and all EMT-related protein primary antibodies were purchased from Abcam Trading Company Ltd (Shanghai, China). GAPDH rabbit monoclonal antibody (AF1186; Beyotime) was regarded as an internal control. Subsequently, these membranes were washed with Tris-buffered saline Tween thrice for 5 min and were incubated with the horseradish peroxidase-conjugated secondary antibodies for 1 h at room temperature. The protein luminescence effect was evaluated via an enhanced chemiluminescence plus with a bioimaging system. Image J software served to scan the gray values.

### 2.6 Cell proliferation assay

After transfection for 24 h, cells at a density of 1,000 cells/well were routinely incubated in 96-well plates. After incubation for 0, 24, 48 and 72 h, 10 μL of cell counting kit 8 (CCK-8; Dojindo, Tabaru, Japan) reagent was supplied to each well. Next the optical density (OD) values were recorded 2 h later using a microplate reader at 450 nm wavelength.

### 2.7 Plate clone formation assay

To prepare cell suspension, trypsin was first used to digest the cells of logarithmic growth. Then at 400 cells/dish, the cell suspension was plated into 60 mm dishes containing 5 mL preculture medium and kept at an atmosphere of 5% CO_2_ at 37°C for 2 weeks. After washing twice with phosphate-buffered saline (PBS), fixing with 4% paraformaldehyde for 30 min and staining with 0.1% crystal violet for 30 min, the number of colonies was counted.

### 2.8 Transwell assay

To evaluate the OC cells’ invasion and migration, 24-well Transwell chambers (Genetimes, Shanghai, China) were adopted. The invasion and migration assay procedures were similar, except that the latter did not perform gelatinization. Briefly, the transfected cells (1 × 10^5^ cells/well) were incubated in 100 μL serum-free RPMI-1640 in the upper chambers, and the lower chambers were supplemented with 500 μL RPMI-1640 plus 10% FBS. Next using the cotton swabs, the residual cells on upper chambers were wiped off when cultured for 24 h. After that, the cells that migrated or invaded to the lower chamber were washed with PBS, fixed and stained for 30 min. Upon being washed with PBS again, the migrated or invaded cells were photographed and counted under a microscope with five random selected fields.

### 2.9 Statistical analyses

Survival curve was plotted utilizing Kaplan–Meier method with log-rank tests for comparison. Comparison of two groups was performed utilizing Student’s *t* test, and one-way analysis of variance served to compare the differences among three or more groups, following a Dunnett *post hoc* test. All statistical analyses were conducted with GraphPad Prism version 6.0 (GraphPad Software, La Jolla, CA, USA) or SPSS version 21.0 (SPSS, Chicago, IL, USA) software. The results of the *in vitro* experiments were expressed as mean value ± standard deviation from triplicate experiments. A *P* < 0.05 was indicated as a statistically significant difference.


**Ethical statement:** Ethical approval was not required. No human and animal assays were used in this study.

## 3 Results

### 3.1 PABPC1 is upregulated in OC and has a connection with poor survival

**Figure 1:**
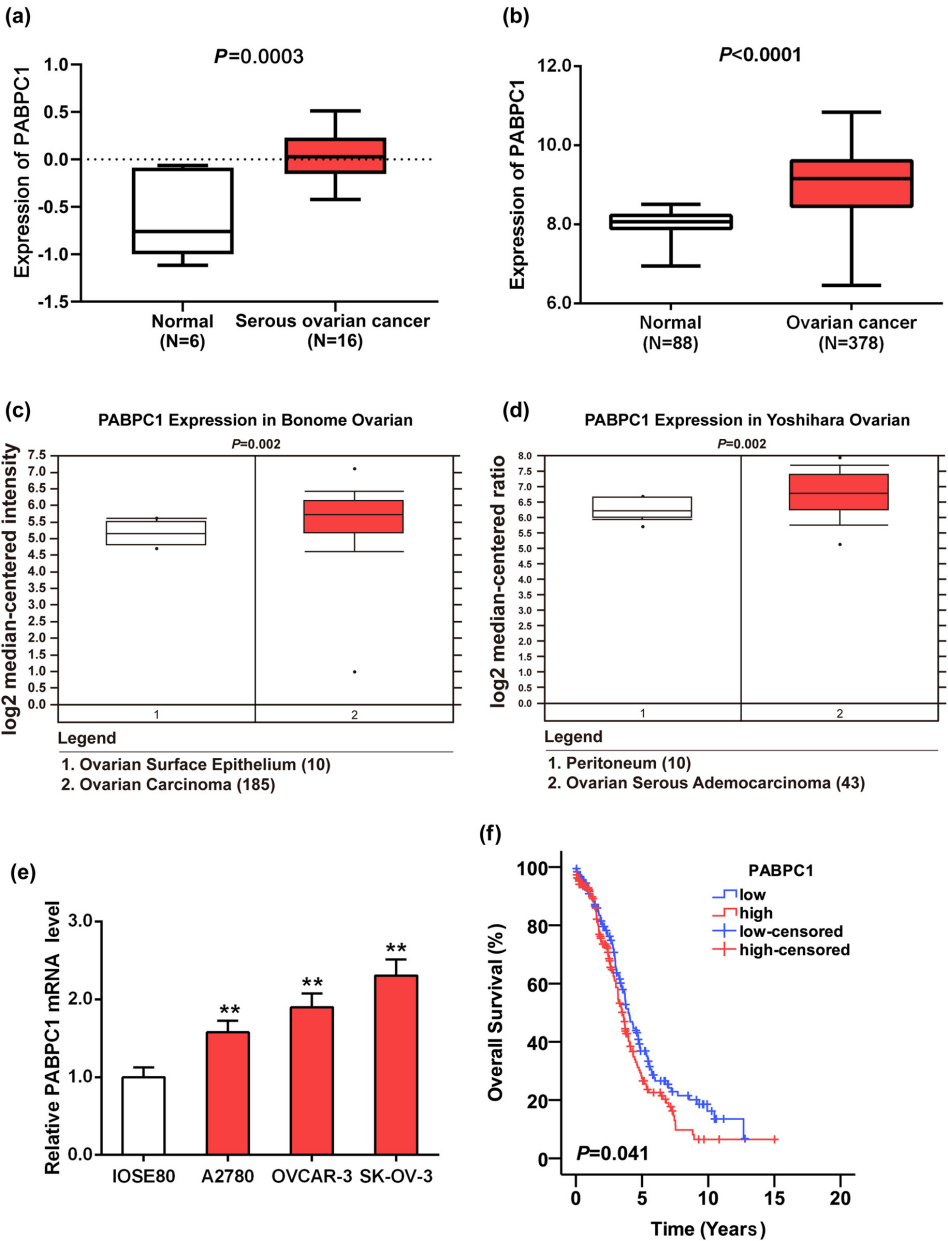
Expression of PABPC1 and Kaplan–Meier curves. High expression of PABPC1 was observed in GSE54388 from GEO database (a), TCGA database and GTEx database (b), and Oncomine database (c and d). (e) mRNA expression of PABPC1 in OC cell lines (A2780, OVCAR-3, SK-OV-3) and normal cell line (IOSE80) was examined using RT-qPCR analysis. **P < 0.01 vs IOSE80 cell line. (f) Kaplan–Meier method was utilized to plot the overall survival curve based upon the TCGA database (*N* = 374).

At first, the expression of PABPC1 was evaluated by bioinformatic analysis using GEO data set GSE54388. As shown in Figure 1a, a significant upregulation was observed in serious OC samples in comparison with normal healthy samples (*P* = 0.0003). Then we analyzed the expression of PABPC1 from TCGA which included 378 OC tissues. Since no normal tissues were observed in the OC-related RNA-Seq data set in TCGA, we downloaded 88 normal ovarian tissue samples from GTEx database as controls. It was shown that PABPC1 expression in OC samples was higher than that in the normal samples (*P* < 0.001; Figure 1b). Moreover, we found that PABPC1 displayed a higher expression in OC tissues based on the Bonome Ovarian data set and Yoshihara Ovarian data set from Oncomine database when compared with nontumor tissues (*P* < 0.01; Figure 1c and d). To validate the observations from bioinformatic analyses, we perform RT-qPCR to examine mRNA expression of PABPC1 in three OC cell lines. A significant upregulation of PABPC1 was shown in all OC cell lines compared to the normal cell line IOSE80 (*P* < 0.01; Figure 1e), which was in agreement with the outcomes by bioinformatic analysis.

The correlation between high/low PABPC1 expression and the overall survival of patients with OC was probed through Kaplan–Meier methods. The curve chart revealed that patients with high PABPC1 levels exhibited a worse overall survival compared with those with low PABPC1 level (*P* = 0.041; Figure 1f).

### 3.2 Detection gain and loss function of PABPC1 efficiency

Based on the previous RT-qPCR assay, we found that, among OC-related cell lines, mRNA expression of PABPC1 was highest in SK-OV-3 cells and lowest in A2780 cells. To make the following tests more optimized, SK-OV-3 was chose to perform the loss of function assay and A2780 was chose as the targeted cells for the next overexpression experiments.

**Figure 2:**
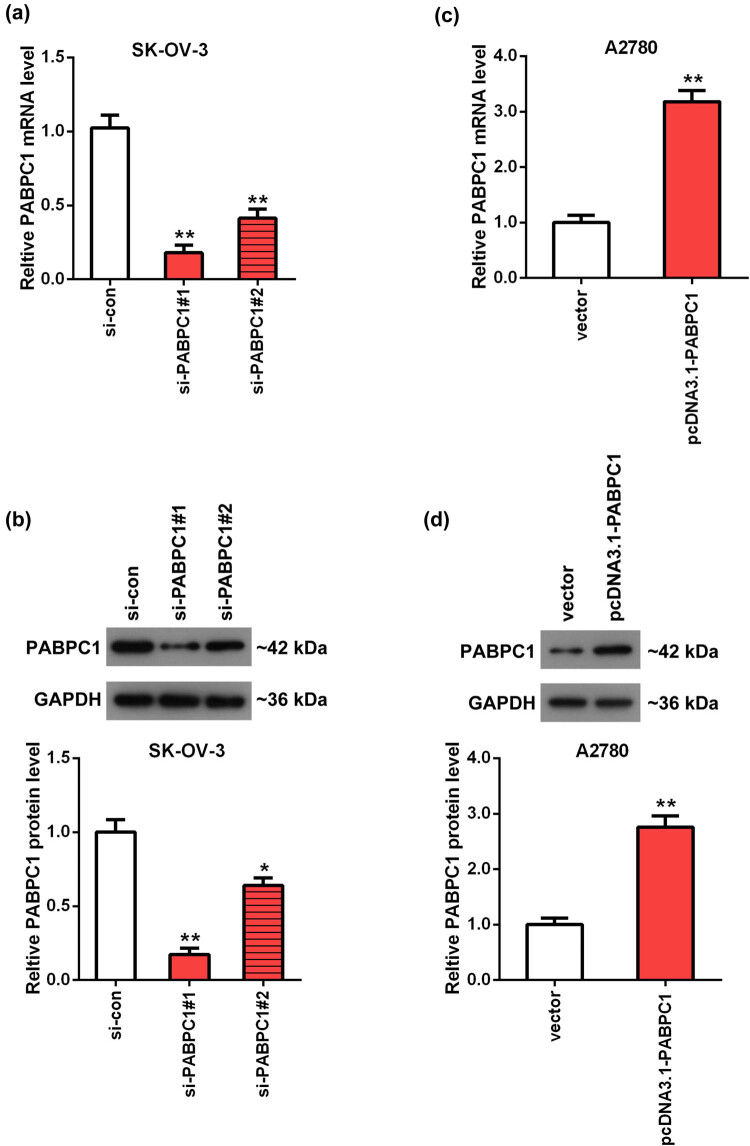
Detection of knockdown and overexpression of PABPC1 efficacy. The mRNA (a and c) and protein (b and d) levels of PABPC1 in SK-OV-3 (a and b) and A2780 (c and d) cells were tested using RT-qPCR (a and c) and Western blot (b and d) analyses. SK-OV-3 cells were transfected with si-PABPC1#1, si-PABPC1#2 and si-con. A2780 cells were transfected with pcDNA3.1-PABPC1 and pcDNA3.1 empty vector. **P < 0.01 vs si-con or vector group.

Efficiency of overexpression and knockdown was measured on the mRNA and protein expression aspects. Upon transfection with si-PABPC1#1 and si-PABPC1#2, the results showed that the mRNA and protein levels of PABPC1 were remarkably reduced in SK-OV-3 cells in contrasts with si-con group (*P* < 0.01; Figure 2a and b). By the way, the sequence of si-PABPC1#1 was selected to carry out the subsequent loss-of-function assay due to its better efficiency than si-PABPC1#2 group. Furthermore, PABPC1 expressions were notably enhanced in A2780 cells after transfecting with pcDNA3.1-PABPC1 in comparison with vector group (*P* < 0.01; Figure 2c and d).

### 3.3 PABPC1 expression impacted OC cell proliferative ability

**Figure 3:**
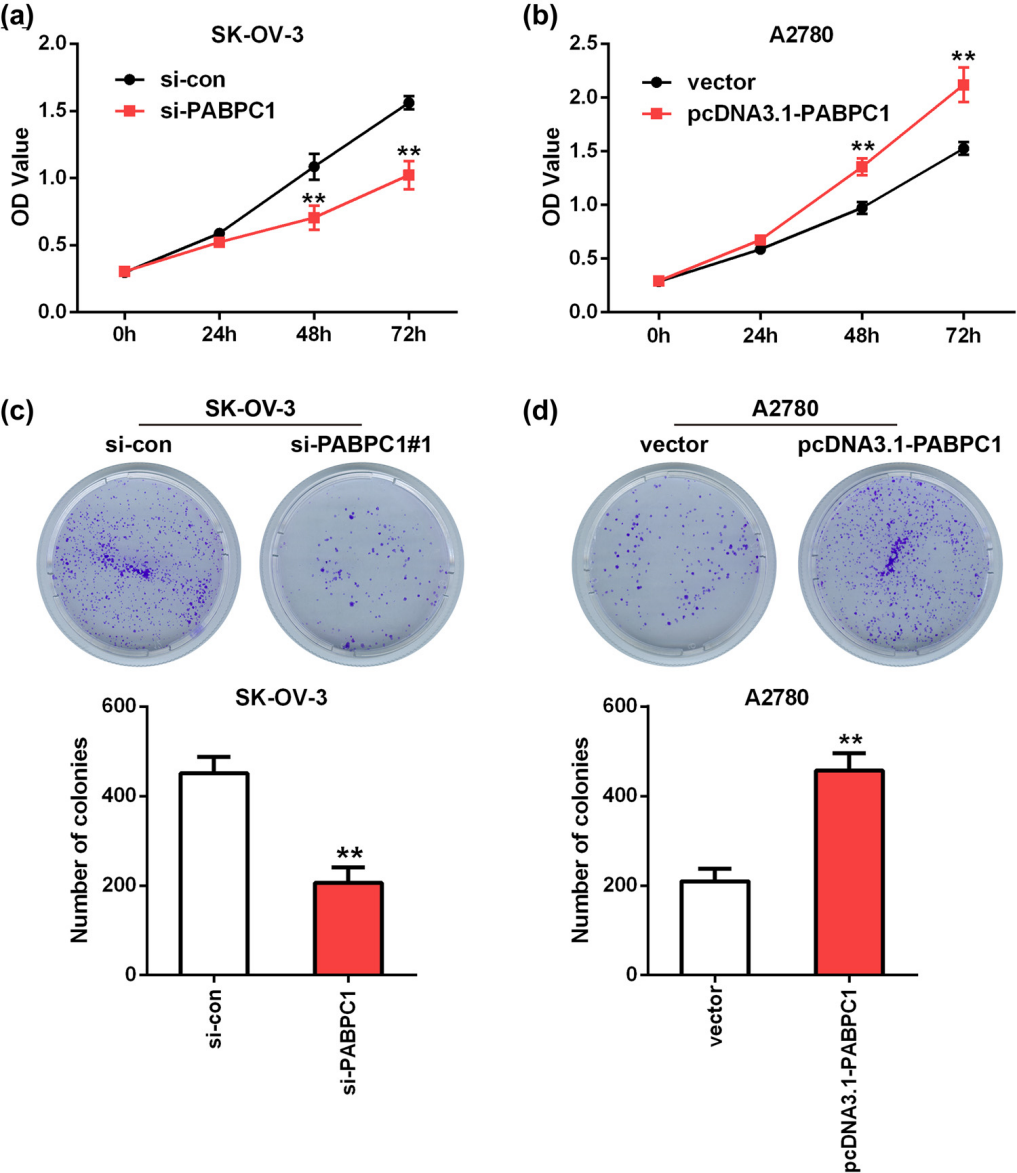
Influences of knockdown and overexpression of PABPC1 on proliferation. The SK-OV-3 cells proliferative (a) and clonogenic (c) capacities were detected utilizing CCK-8 (a) and clone formation (c) assays after depletion of PABPC1. The A2780 cells proliferative (b) and clonogenic (d) capacities were detected utilizing CCK-8 (b) and clone formation (d) assays after upregulation of PABPC1. **P < 0.01 vs si-con or vector group.

To examine the action of PABPC1 on OC cell proliferation, CCK-8 and plate clone formation assays were performed. According to the results of CCK-8 assay, the OD values of SK-OV-3 cells were notably reduced after downregulation of PABPC1 by comparison with control at 48 and 72 h (*P* < 0.01; Figure 3a). In the overexpression tests, the opposite trend appeared, which is based on the evidence that the OD values were remarkably higher in pcDNA3.1-PABPC1 group in comparison with the vector group (*P* < 0.01; Figure 3b).

A similar trend was observed in clone formation assays. As shown in Figure 3c, the visible colonies in si-PABPC1 group was less than that in si-con group in SK-OV-3 cells (*P* < 0.01; Figure 3c). On the contrary, upregulation of PABPC1 in A2780 cells presented a better cloning efficiency when compared with the control (*P* < 0.01; Figure 3d).

### 3.4 PABPC1 expression affected OC cell invasiveness and motility

**Figure 4:**
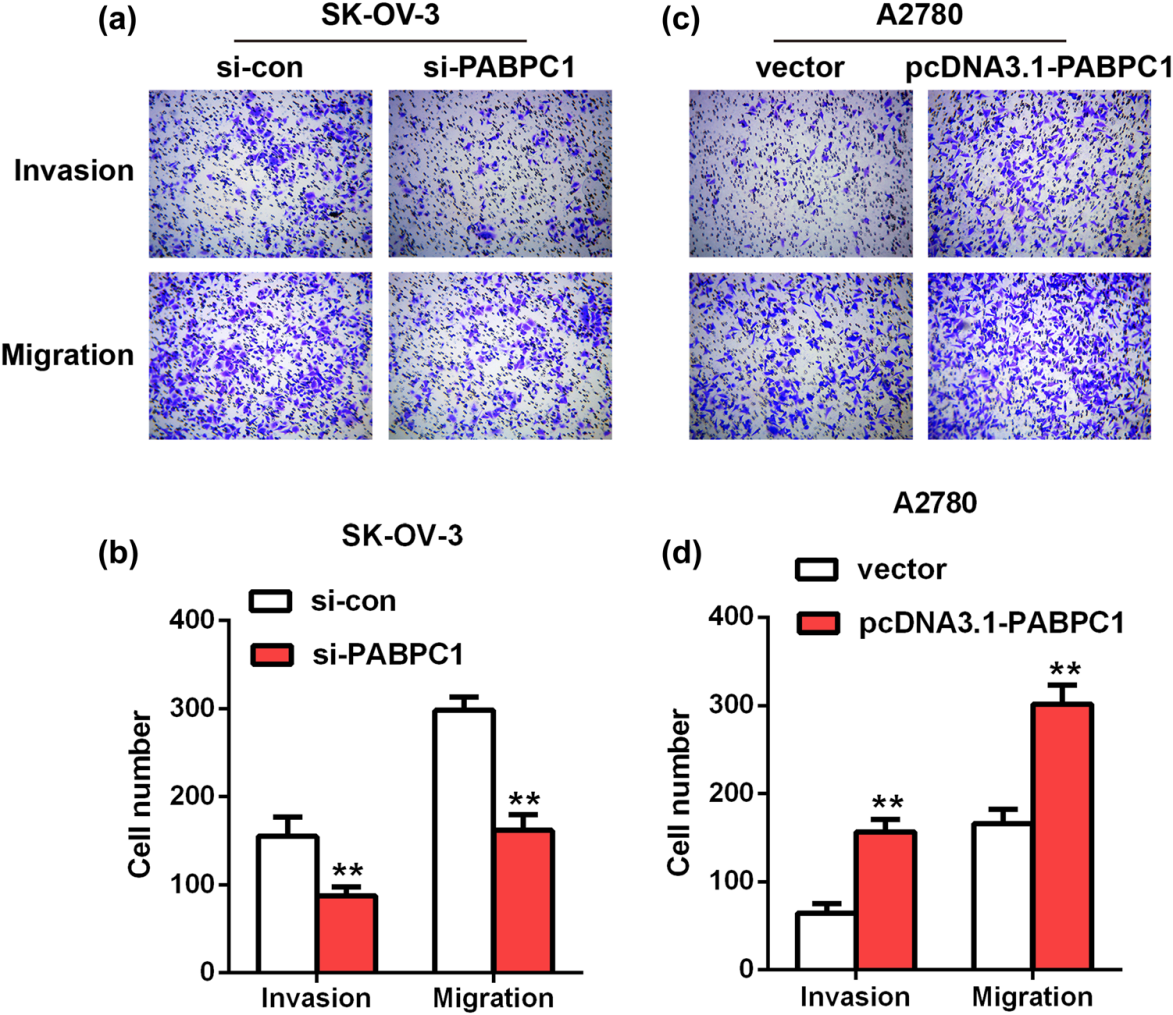
Impacts of downregulation and upregulation of PABPC1 on invasion and migration. The impacts of PABPC1 knockdown on SK-OV-3 cells (a and b) and the impacts of PABPC1 overexpression on A2780 cells (c and d) invasion and migration were measured by Transwell assays.**P < 0.01 vs si-con or vector group.

The action of PABPC1 on the abilities of invasion and migration of OC cells was investigated using Transwell assays. Results of invasion assays revealed that the invaded SK-OV-3 cells in si-PABPC1 group (87.67 ± 17.01) showed a nearly twofold decrease compared with the control (155 ± 38.04; *P* < 0.01; Figure 4a and b). Similarly, the migrated SK-OV-3 cells in si-PABPC1 group (161.67 ± 30.53) were markedly decreased compared with the si-con group (298 ± 25.63; *P* < 0.01; Figure 4a and b). On the other hand, after PABPC1overexpression (156.67 ± 24.03), the invaded number of A2780 cells was more than twice as many as that of the vector group (64.33 ± 18.56; *P* < 0.01; Figure 4c and d). Moreover, the migrated A2780 cells in pcDNA3.1-PABPC1 group (301.33 ± 37.82) were also significantly increased when compared with the vector group (165.67 ± 29.09; *P* < 0.01; Figure 4c and d).

### 3.5 PABPC1 expression was associated with EMT process in OC cells

**Figure 5:**
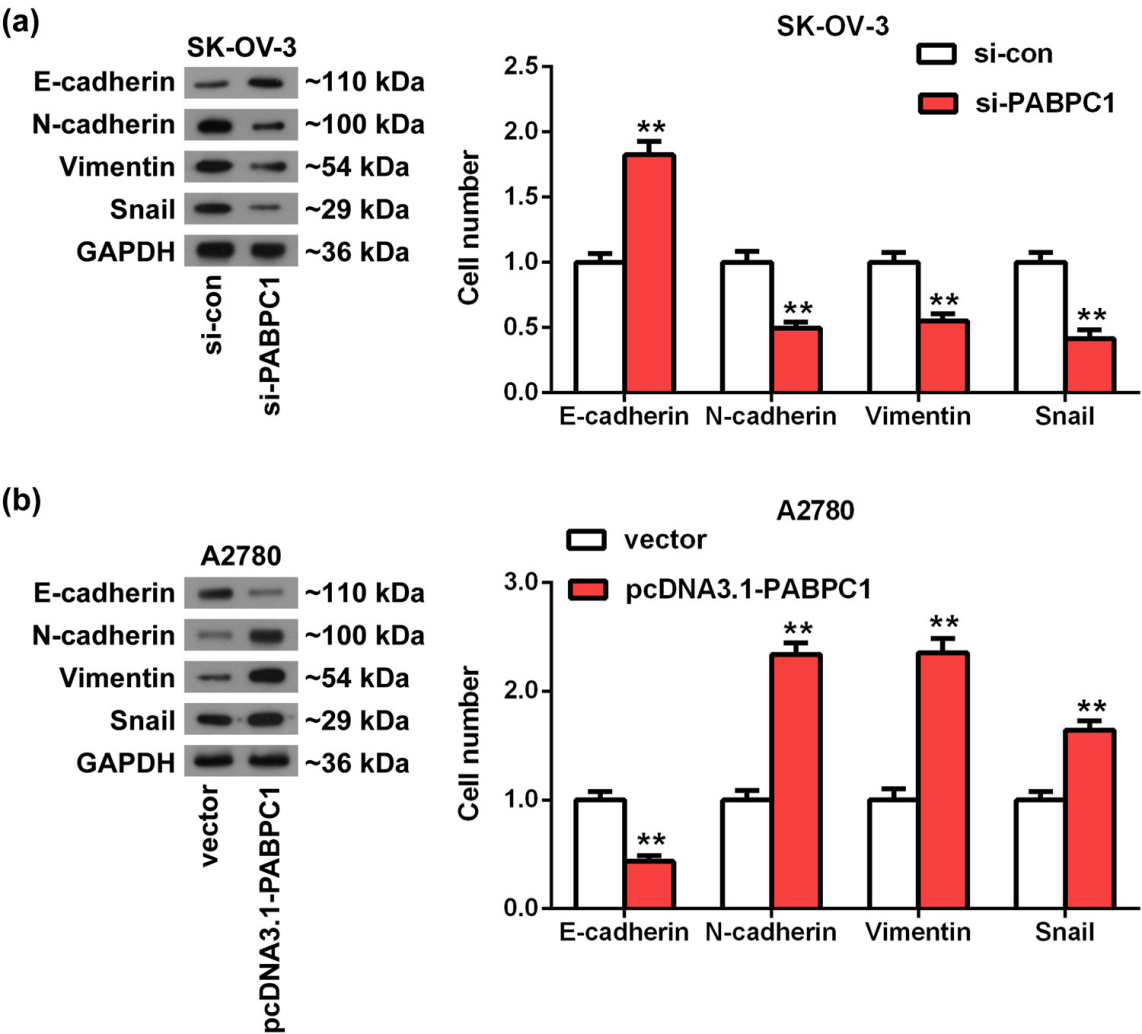
PABPC1 regulated the EMT process. (a and b) Western blot was employed to evaluate the effects of PABPC1 knockdown (a) and PABPC1 overexpression (b) on the protein expression of EMT-related markers in SK-OV-3 (a) and A2780 (b) cells. **P < 0.01 vs si-con or vector group.

Our previous functional experiments indicated that PABPC1 could promote OC cells’ movement and invasion. Considering that the EMT is frequently used to explain how cancer cells acquire aggressiveness [21], we investigated the action of PABPC1 on the EMT-related markers using Western blot analysis. The outcomes illustrated that the protein expression of epithelial marker E-cadherin was notably elevated after depletion of PABPC1 in SK-OV-3 cells compared with the control (*P* < 0.01; Figure 5a), whereas the levels of mesenchymal markers N-cadherin and vimentin and transcription factor snail were all significantly downregulated (*P* < 0.01; Figure 5a). For overexpression of PABPC1, the levels of these markers in A2780 showed the reverse results, as with restrained E-cadherin levels and elevated the levels of N-cadherin, vimentin and snail (*P* < 0.01; Figure 5b).

## 4 Discussion

Although cancer registry statistics revealed an overall decline trend in OC incidence and mortality in Europe, North America and other regions over the past three to four decades, the 5-year relative survival is still less than satisfactory [22]. In recent years, the major treatment of OC remains cytoreductive surgery and platinum-based chemotherapy. Despite most patients of OC initially being platinum sensitive, the majority of them will develop platinum resistance upon several recurrences, which presents a low response to the second-line chemotherapy [23]. Moreover, OC encompasses several heterogeneous subtypes with different clinical phenotypes, molecular features and prognosis [24]. Targeted therapy is expected to be a more effective and less toxic therapeutic strategy for OC. Therefore, with the expectation of providing a new possible view for OC-targeted therapy, we investigated the functional role of PABPC1 on OC cells as well as the potential effect on EMT. Our data showed that PABPC1 serves as an oncogene to facilitate cell proliferative capacity and invasive and migratory abilities by regulating the EMT process in OC.

In this study, we first revealed the upregulation of PABPC1 in OC tissues using bioinformatic analysis and found that high expression of PABPC1 was associated with the poor prognosis in patients with OC, implying that PABPC1 might present a tumorigenic effect on the progression of OC. Furthermore, functional experiments were implemented to determine the action of PABPC1 on the cell viability and aggressiveness using the A2780 and SK-OV-3 cell lines. The results revealed that depletion of PABPC1 dramatically inhibited SK-OV-3 cell viability and invasiveness, while upregulation of PABPC1 in A2780 cells exhibited the opposite results. Noticeably, previous research have also suggested the involvement of PABPC1 in the growth and metastasis of numerous tumors. As reported, highly expressed PABPC1 was observed in gastric carcinoma tissues and could predict poor survival, while downregulation of PABPC1 could induce apoptosis [15]. Similarly, in metastatic duodenal cancer, PABPC1 expression was upregulated in tumor cells, and overexpression of PABPC1 enhanced cell proliferative capacity, colony numbers and metastasis ability [16]. All these data provided evidences supporting that PABPC1 may act as an oncogene involved in the carcinogenesis of OC.

The transition from epithelial cells to motile mesenchymal cells is known as EMT [25], and it is characterized by reduced adhesion, cytoskeletal recombination and increased mobility [26]. EMT has been explored for decades in the development of mammals and is considered to be an important mechanism for tumor progression and metastasis [27]. It can prevent the spread of tumor cells in the early stage or eradicate the existing metastatic cells in advanced stage [28,29]. EMT pathway as a new therapeutic approach for cancer has attracted more and more attention in cancers. In OC, previous studies have reported that microRNA-1271 inhibited the EMT process [30], whereas miR-9 promoted the EMT [31]. Moreover, overexpression of TEL1 in SK-OV-3 cells could reverse the EMT process [32] and knockdown of HOXB-AS3-inhibited epithelial OC cell EMT [33]. These reports prompted us to probe whether PABPC1 is connected with the EMT process in OC. To gain insight into this underlying mechanism of action, we detected EMT relative protein levels including E-cadherin, N-cadherin, vimentin and snail. Western blot revealed that reducing PABPC1 levels in SK-OV-3 cells could significantly suppress the EMT action, as evidenced by raising E-cadherin levels and reducing N-cadherin, vimentin and snail levels, whereas increasing PABPC1 expression got the reverse outcomes. These results suggested that PABPC1 as a cancer-promoting gene facilitated the growth and invasiveness of OC cells partly by regulating the EMT. As we know, elucidating the molecular mechanism of the regulation process of EMT and exploring the treatment targeting molecule of EMT are the key issues in the study of the EMT mechanism in malignant tumors [34,35]. Therefore, further investigations will be needed on the correlation of PABPC1 expression and EMT in OC.

The present study has limitations. One limitation of this study is that we only analyzed PABPC1 expression in OC and its relationship with the overall survival using the online data sets. Hence, the prognostic significance of PABPC1 in relevant clinical specimens has to be assessed to confirm its potential involvement. Another limitation is that the functional experiments in this study were obtained from human OC cell lines *in vitro*. The *in vivo* functions of PABPC1 in OC development in the pathogenesis of OC need to be further explored.

In summary, this study demonstrated that PABPC1 was significantly upregulated in OC cells and had a connection with poor survival rate for patients with OC. Functionally, our work suggested that PABPC1 plays a promotional effect on the cells’ proliferation, invasion and migration in OC partly through regulating the EMT process. These data imply that PABPC1 may serve as a biological carcinogene in the development of OC, which provides a new insight of research and therapeutic interventions for OC.
